# National variation in United States sepsis mortality: a descriptive study

**DOI:** 10.1186/1476-072X-9-9

**Published:** 2010-02-15

**Authors:** Henry E Wang, Randolph S Devereaux, Donald M Yealy, Monika M Safford, George Howard

**Affiliations:** 1 Department of Emergency Medicine, University of Alabama at Birmingham, Birmingham, Alabama, USA; 2 Department of Emergency Medicine, University of Pittsburgh, Pittsburgh, Pennsylvania, USA; 3 Department of Internal Medicine, Division of Preventive Medicine, University of Alabama at Birmingham, Birmingham, Alabama, USA; 4 Department of Biostatistics, University of Alabama at Birmingham, Birmingham, Alabama, USA

## Abstract

**Background:**

The regional distribution of a disease may provide important insights regarding its pathophysiology, risk factors and clinical care. While sepsis is a prominent cause of death in the United States (US), few studies have examined regional variations with this malady. We identified the national variation in sepsis deaths in the US. We conducted a descriptive analysis of 1999-2005 national vital statistics data from the National Center for Health Statistics summarized at the state-level. We defined sepsis deaths as deaths attributed to an infection, classified according to the International Classification of Diseases, Version 10. We calculated national and state age-adjusted sepsis-attributed mortality rates.

**Results:**

National age-adjusted sepsis mortality was 65.5 per 100,000 persons (95% CI: 65.8 - 66.0). State level sepsis mortality varied more than two-fold (range 41 to 88.6 per 100,000 persons; median 60.8 per 100,000, IQR 53.9-74.4 per 100,000). A cluster extending from the Southeastern to the mid-Atlantic US encompassed states with the highest sepsis mortality.

**Conclusions:**

Sepsis mortality varies across the US. The states with highest sepsis mortality form a contiguous cluster in the Southeastern and mid-Atlantic US. These observations highlight unanswered questions regarding the characteristics and care of sepsis.

## Background

Infections may lead to death by triggering systemic inflammation (sepsis), subsequent organ dysfunction and shock [1,2]. Each year in the United States (US), sepsis results in 570,000 emergency department visits and affects over 750,000 hospitalized patients. There are approximately 200,000 US sepsis deaths annually, underscoring the public health magnitude and importance of this process [3,4].

While prior studies characterize the epidemiology of sepsis in the US, few studies have examined regional variations with this malady [3,5-7]. A prominent example of regional disease variation is the "Stroke Belt," a cluster of excess cerebrovascular deaths in the Southeastern US [8-14]. Regional disease variation is important, pointing to potential differences in patient characteristics, pathogen exposure, disease susceptibility, health behaviors, pre-existing medical conditions, socioeconomic resources, genetic polymorphisms, healthcare resources or quality of care [11,12,14].

Mortality is a function of susceptibility (attack rate) and case fatality. While the scientific community has focused primarily on the acute care of sepsis, there have been only limited efforts to prevent or to identify individuals most susceptible to sepsis, which could vary regionally [1,12]. For example, systematic regional differences in the quality of sepsis care could alter sepsis mortality. A better understanding of the geographic patterns of sepsis mortality could lead to new insights regarding the diagnosis, treatment and prevention of sepsis.

We sought to characterize the US regional variation in sepsis deaths by examining the national distribution of deaths attributed to infection. We hypothesized that there would be substantial variation in sepsis mortality across the US.

## Methods

### Study Design

We conducted a descriptive analysis using mortality data from the National Center for Health Statistics (NCHS). The Institutional Review Board of the University of Alabama at Birmingham approved the study without the requirement for informed consent from patients.

### Study Setting

We studied deaths attributed to infection in the continental US, including the District of Columbia.

### Sources of Data

For this study we used the National Center for Health Statistics' Compressed Mortality File (CMF), which contains data on the age, race, sex, year and causes of all US deaths [15]. We chose the CMF for this analysis because it represents the only data set aggregating US death incidence and geographic distribution for different disease groups. While individual states often compile more detailed information on individual deaths (for example, the specific hospital or location of death), development of national geographic perspectives would have required combining multiple data sets.

US Census Bureau population estimates supplement the CMF data. We used the most recent CMF death information, averaged across a seven-year period (1999-2005) to stabilize the estimates. We chose not to assess variations in sepsis hospitalization because currently available data lacked adequate resolution for meaningful geographic analyses [3,5,7].

### Study Population

We defined sepsis death as death attributed to an infection. We chose this approach because of the large overlap between sepsis and infection deaths. Prior studies using administrative and mortality data have characterized only hospitalizations or deaths attributed to sepsis or septicemia [5,16]. However, if we similarly examined only deaths specifically attributed to sepsis (ICD-10 A40-A41), we would have underestimated the true number of cases. For example, in the case of pneumonia with associated sepsis, the data set may have attributed the death to pneumonia rather than sepsis. Death from infection often occurs through organ failure; the pattern of infection plus organ dysfunction is consistent with international consensus definitions of sepsis [1]. We aggregated all deaths due to infection as done in prior studies of sepsis [3,6,7].

The CMF classifies deaths for 1999-2005 using the International Classification of Diseases, Tenth Revision (ICD-10), grouping cause of death into 113 broad categories. We identified cause of death categories related to infection, including septicemia, respiratory (-e.g., pneumonia), abdominal and gastrointestinal (-e.g., appendicitis, diverticulitis), cardiac (-e.g., endocarditis), kidney and genitourinary (-e.g., pyelonephritis, pelvic inflammatory disease), neurologic (meningitis) and other infections. (Table 1) We also included influenza (ICD-9 487 - corresponding to ICD-10 J10-J11), acute bronchitis and bronchiolitis (ICD-9 466 - corresponding to ICD-10 J20-J21), and pneumonitis due to solids and liquids (ICD-9 507 - corresponding to ICD-10 J69). These categories closely aligned with previously used ICD-9 based classifications of severe sepsis applied to hospital discharge and emergency department data [3,6,7].

**Table 1:** Infection-attributed deaths and corresponding infection subgroups.

Cause of Death Category	ICD-9 Codes	ICD-10 Codes	Infection Subgroup
Salmonella infections	002-003	A01-A02	Abdominal
Shigellosis and amebiasis	004, 006	A03, A06	Abdominal
Certain other intestinal infections	007-009	A04, A07-A09	Abdominal
Respiratory tuberculosis	010-012	A16	Respiratory
Other tuberculosis	013-018	A17-A19	Respiratory
Whooping cough	33	A37	Respiratory
Scarlet fever and erysipelas	034.1-035	A38, A46	Other
Meningococcal infection	36	A39	Neurological
Septicemia	38	A40-A41	Septicemia
Syphilis	090-097	A50-A53	Kidney/Genitourinary
Acute poliomyelitis	45	A80	Other
Arthropod-borne viral encephalitis	062-064	A83-A84, A85.2	Neurological
Measles	55	B05	Other
Viral hepatitis	70	B15-B19	Abdominal
Human immunodeficiency virus (HIV) disease	042-044	B20-B24	Other
Malaria	84	B50-B54	Other
Other and unspecified infectious and parasitic diseases and their sequelae	001, 005, 020-032, 037, 039-041, 046-054, 056-061, 065-066, 071-083, 085-088, 098-134, 136-139, 771.3	A00, A05, A20-A36, A42-A44, A48-A49, A54-A79, A81-A82, A85.0-A85.1, A85.8, A86-B04, B06-B09, B25-B49, B55-B99	Other
Meningitis	320-322	G00, G03	Neurological
Acute and subacute endocarditis	421	I33	Cardiac
Diseases of pericardium and acute myocarditis	420, 422-423	I30-I31, I40	Cardiac
Influenza	487	J10-J11	Respiratory
Pneumonia	480-486	J12-J18	Respiratory
Acute bronchitis and bronchiolitis	466	J20-J21	Respiratory
Unspecified acute lower respiratory infection	---	J22	Respiratory
Pneumonitis due to solids and liquids	507	J69	Respiratory
Diseases of Additional File 1 - Appendices	540-543	K35-K38	Abdominal
Cholelithiasis and other disorders of gallbladder	574-575	K80-K82	Abdominal
Infections of kidney	590	N10-N12, N13.6, N15.1	Kidney/Genitourinary
Inflammatory diseases of female pelvic organs	614-616	N70-N76	Kidney/Genitourinary

Sepsis-attributed deaths defined as deaths due to infection. The National Center for Health Statistics (NCHS) uses 113 causes of death categories to classify each death. Adopted from "NCHS 113 Selected Causes of Death"[31].

For this study we used the CMF "underlying cause of death" data set, which identifies a single initiating disease or injury event leading to death [15]. Customarily, the underlying cause of death is based upon death certificate documentation. For example, in the case of a patient hospitalized for pneumonia but later developing sepsis and death, the death record may attribute the death to pneumonia. When physicians enter more than one cause or condition, the CMF uses the sequence of listed conditions, provisions of the International Classification of Diseases, and associated selection rules and modifications to classify the underlying cause of death [15]. We did not use the contrasting CMF "multiple cause of death" data set because of the potential for misattribution.

We included infection-attributed deaths for all individuals ≥ 15 years old during the period 1999-2005. We included deaths in individuals aged 15-19 years because the CMF uses a single reference standard population for ages 15-24; inclusion of the 15-19 year group is necessary for age-adjustment. We excluded individuals <15 years because the epidemiology of sepsis differs for this age group [17]. We excluded individuals with unknown age.

### Statistical Analysis

We used an analytical approach similar to prior studies of stroke death clusters [9,10,13]. We used age-adjusted mortality rates provided by CMF, which adjusts relative to intercensal (1999), actual (2000) or postcensal (2001 to 2005) US Census population estimates. We determined the age-adjusted sepsis/infection-attributed mortality rate nationally and for each state referenced to the 2000 US population. We used a similar approach for African Americans and Whites. We determined unadjusted national and state sepsis/infection-attributed mortality for different age categories (15-24, 25-44, 45-65, and ≥ 65 years). We also calculated age-adjusted mortality for each infection subgroup. The CMF defined the location of death as the person's place of residence. We graphically depicted the geographic distributions across the US. We analyzed all data using Stata 10.1 (Stata, Inc., College Station, Texas) and Excel (Microsoft, Inc., Redmond, Washington).

## Results

During 1999-2005, among persons ≥ 15 years old there were 1,041,404 deaths due to infection, corresponding to a national age-adjusted mortality rate of 65.9 deaths per 100,000 persons (95% CI: 65.8-66.0). (Table 2) Most deaths were attributed to respiratory infections, septicemia and abdominal and gastrointestinal infections. (Table 3)

**Table 2:** Sepsis attributed deaths, United States, 1999-2005

State	Sepsis- Attributed Deaths 1999-2005	Population 2000	Crude Sepsis-Attributed Mortality (Annual Deaths per 100,000)	Age-Adjusted Sepsis-Attributed Mortality (Annual Deaths per 100,000; 95% CI)
Minnesota	11,907	4,622,379	42.9	41.0 (40.2 - 41.8)
North Dakota	2,057	597,853	57.3	44.9 (42.7 - 47.1)
Alaska	853	568,616	25.0	46.0 (43.7 - 48.3)
New Hampshire	3,214	1,185,137	45.2	46.4 (44.8 - 48.0)
Oregon	9,624	3,283,846	48.8	46.5 (45.5 - 47.5)
Vermont	1,703	582,797	48.7	47.2 (44.9 - 49.5)
Nebraska	5,467	1,588,076	57.4	50.0 (48.6 - 51.4)
Wisconsin	16,520	5,050,218	54.5	50.3 (49.5 - 51.1)
Washington	16,229	5,610,002	48.2	50.9 (50.1 - 51.7)
Idaho	3,518	1,210,933	48.4	50.9 (49.3 - 52.6)
South Dakota	2,682	699,723	63.9	51.8 (49.6 - 54.0)
Montana	2,982	854,145	58.2	53.3 (51.3 - 55.3)
New Mexico	5,138	1,682,975	50.9	53.9 (52.5 - 55.3)
Iowa	11,605	2,738,125	70.6	55.7 (54.6 - 56.8)
Maine	4,653	1,232,075	62.9	55.9 (54.2 - 57.6)
California	94,587	31,544,211	50.0	55.9 (55.6 - 56.2)
Florida	64,486	15,830,981	67.9	56.5 (56.0 - 57.0)
Utah	5,075	2,000,507	42.3	56.7 (55.4 - 58.1)
Michigan	31,031	9,211,209	56.1	56.7 (56.1 - 57.3)
Colorado	11,276	4,115,392	45.7	56.8 (55.9 - 57.7)
Kansas	9,514	2,486,519	63.8	56.8 (55.6 - 58.0)
Hawaii	4,216	1,153,656	60.9	57.8 (56.0 - 59.6)
Wyoming	1,507	464,375	54.1	57.8 (55.0 - 60.7)
Rhode Island	4,199	1,001,684	69.9	59.7 (57.8 - 61.7)
Arizona	17,673	4,944,262	59.6	59.9 (59.0 - 60.8)
Ohio	41,051	10,560,870	64.8	61.7 (61.1 - 62.3)
Indiana	21,115	5,658,032	62.2	62.2 (61.4 - 63.0)
West Virginia	7,611	1,731,920	73.2	64.8 (63.3 - 66.4)
Texas	65,887	19,423,021	56.5	67.4 (66.9 - 67.9)
Missouri	23,510	5,256,289	74.5	69.1 (68.2 - 70.0)
Pennsylvania	58,013	11,622,733	83.2	69.3 (68.7 - 69.9)
Oklahoma	14,064	3,214,010	72.9	69.9 (68.7 - 71.1)
Illinois	48,771	11,518,522	70.6	71.0 (70.4 - 71.6)
Connecticut	15,634	3,210,413	81.2	71.3 (70.1 - 72.5)
Massachusetts	29,186	6,021,153	80.8	73.1 (72.2 - 74.0)
New York	81,756	17,824,477	76.4	73.9 (73.4 - 74.4)
Nevada	7,413	1,985,276	62.2	74.1 (72.6 - 75.7)
Kentucky	16,517	3,812,718	72.2	74.4 (73.3 - 75.5)
South Carolina	16,070	3,805,040	70.4	74.4 (73.3 - 75.5)
Virginia	27,345	6,779,988	67.2	75.8 (75.0 - 76.6)
North Carolina	33,061	7,708,376	71.5	76.3 (75.5 - 77.1)
Alabama	19,304	4,154,300	77.4	76.6 (75.5 - 77.7)
Arkansas	12,676	2,506,037	84.3	77.4 (76.0 - 78.8)
Delaware	3,445	749,403	76.6	77.6 (75.0 - 80.2)
Tennessee	24,539	5,391,291	75.9	78.3 (77.3 - 79.3)
New Jersey	39,688	7,930,108	83.4	79.4 (78.6 - 80.2)
Mississippi	12,820	2,603,847	82.1	83.7 (82.3 - 85.1)
Louisiana	19,143	4,076,687	78.3	83.7 (82.6 - 84.9)
Georgia	32,420	7,800,211	69.3	85.0 (84.2 - 85.8)
Maryland	24,753	5,011,312	82.3	88.6 (87.5 - 89.7)
District of Columbia	3,896	557,759	116.4	122.7 (119.0 - 126.5)

**TOTAL**	**1,041,404**	**265,173,485**	**65.5**	**65.9 (65.8 - 66.0)^†^**

Includes deaths due to an infection, age ≥ 15 years. Listed in order of ascending age-adjusted sepsis-attributed mortality. *Unreliable estimate due to limited African American population. ^†^Among state-level estimates, minimum 41.0 per 100,000, maximum 122.7 per 100,000, median 61.7 per 100,000 (interquartile range 53.9-74.4 per 100,000).

**Table 3:** Sepsis attributed deaths, stratified by infection subgroup.

	State-Level Mortality		
**Infection Subgroup**	**Minimum** **(deaths/100,000)**	**Maximum** **(deaths/100,000)**	**Median (IQR)** **(deaths/100,000)**

Respiratory	23.9	47.6	35.3 (33.0 - 39.3)
Septicemia	3.6	26.0	14.0 (8.6 - 19.3)
Abdominal/Gastrointestinal	3.1	8.0	4.9 (4.2 - 5.6)
Kidney/Genitourinary	0.2	0.9	0.4 (0.3 - 0.6)
Cardiac	0.6	1.8	0.9 (0.8 - 0.9)
Neurological	0.2	0.5	0.3 (0.3 - 0.4)
Other	3.2	56.0	6.1 (4.3 - 9.6)

State-level mortality per 100,000 persons ages ≥ 15 years, United States, 1999-2005. Includes contiguous United States and District of Columbia.

Overall, the District of Columbia had the highest age-adjusted sepsis mortality (122.7 annual deaths per 100,000). Excluding the District of Columbia, there was an over two-fold variation in age-adjusted sepsis mortality across US states (range 41 to 88.6 per 100,000 persons; median 60.8, IQR: 53.9-74.4).

**Figure 1:**
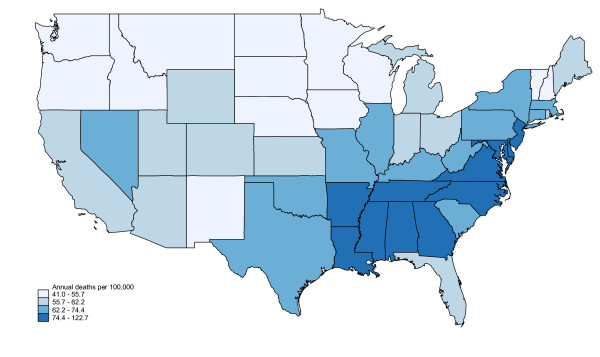
**Regional variation in sepsis mortality, United States, 1999-2005**. Excludes Alaska and Hawaii.

A cluster of 11 adjacent states in the Southeastern and mid-Atlantic US (Arkansas, Louisiana, Mississippi, Alabama, Georgia, Tennessee, North Carolina, Virginia, Maryland, Delaware and New Jersey) contained the highest infection death rates. (Figure 1) Sepsis mortality in this "belt" exceeded sepsis mortality in non-belt regions (80.2 vs. 62.2 per 100,000; incidence rate ratio 1.291, 95% CI: 1.287-1.297).

**Figure 2:**
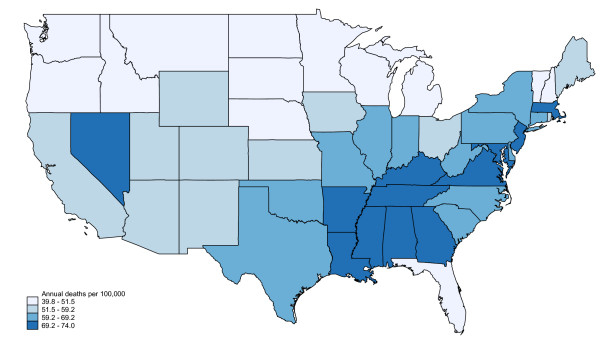
**Regional variation in sepsis mortality, Whites only, United states, 1999-2005**. Excludes Alaska and Hawaii.

**Figure 3:**
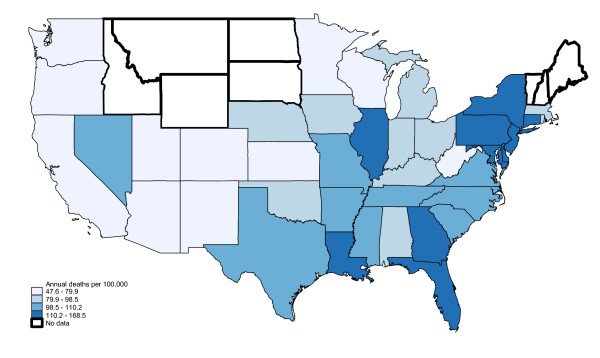
**Regional variation in sepsis mortality, African Americans only, United States, 1999-2005**. Excludes Alaska and Hawaii. Bold borders indicate states where reliable age-adjusted mortality rates could not be calculated.

African Americans exhibited higher overall sepsis mortality than Whites (109.1 vs. 61.0 per 100,000; incidence rate ratio 1.79, 95% CI: 1.78-1.780). The distribution for Whites was similar to overall mortality, with a geographic cluster of high sepsis mortality in the Southeast and mid-Atlantic states. (Figures 2, 3) However, the regional pattern for African Americans differed, with a cluster in the Northeastern US (Maryland, Delaware, Pennsylvania, New Jersey, New York and Connecticut) and several other isolated states (Illinois, Louisiana, Georgia, Florida).

Stratified by age group, unadjusted sepsis/infection mortalities were (per 100,000): 15-24 years, 1.73 (95% CI: 1.68-1.78); 25-44 years, 13.89 (13.80-13.99); 45-64 years, 32.98 (32.81-33.14); ≥ 65 years, 297.05 (296.38-297.73). The regional patterns for each age stratum exhibited similar clustering in the Southeast and Mid-Atlantic states. (See additional file 1 - Appendices 2A-2D) Male (79.30, 95% CI: 79.12-79.49) and female (54.00, 95% CI: 53.85-54.15) sepsis mortality exhibited similar regional distributions. (See additional file 1 - Appendices 3A-3B) Sepsis clusters persisted for the respiratory, septicemia and "other" infection subgroups. (See additional file 1 - Appendices 4A-4G)

## Discussion

The regional variation of a disease may have important implications for its diagnosis and care. For example, sudden cardiac arrest incidence varies two-fold across the US, potentially reflecting differences in population characteristics and public systems of emergency care [8]. Stroke death is highest in the Southeast US, raising questions regarding the stroke susceptibility, health behaviors and systems of medical care [9-14]. Only limited data describe the regional distribution of sepsis [3,5].

We observed a more than two-fold variation in the infection-attributed mortality in the US. The 11 states with the highest sepsis mortality comprised a contiguous cluster in the Southeastern and Mid-Atlantic US. Assuming the equivalence of sepsis and infection-attributed deaths, the increased sepsis mortality in this cluster (80.1 vs. 61.9 deaths per 100,000 in other regions) translates to over 8,500 excess adult sepsis deaths each year in the US.

The reasons for these observations remain unclear but may involve variations in the patients, environment or patterns of care. For example, the treatment of sepsis is often complex, involving the administration of intravenous fluids, antibiotics and vasopressors [4,18]. Regional sepsis mortality variations could reflect differences in the execution of sepsis treatment protocols. Regional differences in medical comorbidities, health behaviors, diet, socioeconomic status, genetics or environmental exposures may potentially alter the risk of sepsis [11,12]. Obesity is highest in the Southeastern US, and sepsis severity is higher in obese individuals, suggesting a potential contributory role [19-24]. Answers to these and other key questions could innovate sepsis treatment and prevention strategies, potentially reducing sepsis death and healthcare expenditures.

Our results provide interesting initial perspectives. For example, the regional distribution of infection deaths remained stable across age and sex strata, suggesting that age and sex are not contributors to regional variation. In contrast, the geographic distribution differed between African Americans and Whites, suggesting that racial differences may partially explain sepsis mortality variations. Prior studies of the sepsis epidemiology have used primarily hospital discharge data with inadequate scope or clinical detail to answer these questions [3,5]. Appropriate answers would require study with a national population-based cohort encompassing knowledge of subjects' baseline characteristics and identification of subsequent sepsis events. Our observations also highlight that population-based studies limited to smaller regions may not result in nationally generalizable inferences.

The most unexpected observation was the similarity between the observed sepsis death cluster and the US "Stroke Belt." While defined in different ways, the Stroke Belt generally refers to a region of increased stroke mortality encompassing Mississippi, Alabama, Georgia, Tennessee, Kentucky, North Carolina and South Carolina [9-13]. Within the Belt a "Stroke Buckle" encompassing the North Carolina, South Carolina and Georgia costal regions contains the highest death rates. First identified in the 1930s, the pattern of excess deaths persists today despite secular trends in overall and race-stratified stroke mortality[9,10,13]. The Stroke Belt has spawned key hypotheses regarding the pathophysiology of and risk factors for cerebrovascular disease, including medical comorbidities, lifestyle, diet, socioeconomic status, genetics, differing responses to medications and environmental exposures [11,12]. The overlap between the Stroke Belt and our observed sepsis cluster could point to unidentified similarities in the pathophysiology, patient characteristics or medical care of these conditions.

There are key differences between this study and prior sepsis epidemiology descriptions. Our estimate of 65.5 infection deaths per 100,000 contrasts with Martin, et al.'s estimate of 43.9 per 100,000. However, Martin, et al. used sampled data from National Hospital Discharge Survey and limited cases to those with ICD-9 sepsis diagnosis codes, potentially missing infection-related deaths not coded as sepsis [5]. Angus, et al.'s study of combined statewide hospital discharge data (Florida, Maryland Massachusetts, New Jersey, New York, Virginia, and Washington) estimated a higher mortality (approximately 85.8 per 100,000), but their broad use of discharge diagnoses may have misattributed selected deaths to infection [3].

Melamed and Sorvillo examined secular trends in sepsis mortality using CMF multiple cause of death data set, classifying sepsis deaths as instances where any of the four causes of death included ICD-10 septicemia [16]. They did not include other infection groups. Our approach differs in the use of a single underlying cause of death complemented by a broader sepsis definition. While our estimate of 65.5 sepsis deaths per 100,000 is higher than Melamed's estimate of 50.5 per 100,000, we included only individuals ≥ 15 years old. When we repeated our analysis using Melamed's approach but limited to individuals age ≥ 15 years, we observed a sepsis mortality of 62.6 per 100,000 as well as the same regional sepsis "belt." This observation supports the robustness of our approach.

Limitations of this analysis include the use of public death records. Listed causes of death are subject to classification or misattribution bias, which could affect our results [25-30]. We could not use conventional definitions of sepsis. We could not ascertain if secondary infections played prominent roles in the death of individual cases. While we used the CDC's existing cause of death categories, this taxonomy may have missed selected infections such as peritonitis, pyothorax, abscesses or unspecified infections. For analytic purposes we combined all infections together, but select patients may have responded differently to individual infections. It is unclear how these biases may have altered our observations. We did not include deaths of individuals <15 years or with unknown age. We note that there were only 56 deaths with unknown age.

We did not formally validate the accuracy of death records for identifying sepsis; this is the objective of a separate effort using adjudicated death records. However, when we repeated the analysis using Melamed's strategy with the CMF multiple cause of death data, we observed similar results, suggesting robustness of our approach. As discussed previously, we did not evaluate regional variations in sepsis hospitalizations because of the lack of appropriate data sets.

While our observed cluster appears to exclude South Carolina and Kentucky (two prominent representatives of the Stroke Belt) these states fell on the upper quartile cutoff (74.4 per 100,000) and could be included with this group. While stroke and infection may conceivably coexist in a patient, we used the CMF underlying cause of death data, precluding the possibility of confounding as the cause of stroke/sepsis geographic overlap.

We selected states as the unit of analysis in order to provide clearer national perspectives for this initial effort. Additional insights may have resulted from smaller geographic units (counties, census tracts). Also, geographic boundaries may not align with state boundaries. For example, heightened sepsis mortality in the Appalachian Mountains would have affected sepsis mortality estimates in many high risk states.

We included only deaths for individuals age ≥ 15 years in this study. We would expect different mortality patterns for children since the sepsis epidemiology differs in this age group[17]. Due to their relatively sparse numbers, we did not separately examine sepsis patterns among Asians/Pacific Islanders and American Indians. Our study describes the regional distribution of those dying from sepsis but does not characterize survivors. We did not have sociodemographic or hospitalization information on each patient. While we did not formally evaluate longitudinal trends, we found similar regional patterns for each year of 1999-2005.

## Conclusions

Sepsis mortality varies across the US. The states with the highest sepsis mortality form a contiguous cluster from the Southeastern to mid-Atlantic US. These observations highlight unanswered questions regarding the characteristics and care of sepsis.

## Competing interests

The authors declare that they have no competing interests.

## Authors' contributions

HEW, RDS, DMY, GH and MMS conceived the study. HEW and RDS obtain the data and carried out the analysis. HEW drafted the manuscript, and all authors contributed significantly to its critical revision and editing. All authors read and approved the final manuscript.

## Supplementary Material

Additional file 1 **APPENDICES, Wang, et al.: National Variation in United States Sepsis Mortality: a Descriptive Study**. Appendices 1A-1D - Age-stratified maps; Appendices 2A-2B - Sex-stratified maps; Appendices 3A-3G - Infection group-stratified maps. Click here for file
