# Supplementary material for: National variation in United States sepsis mortality: a descriptive study
Source: Int J Health Geogr. 2010 Feb 15;9:9. doi: 10.1186/1476-072X-9-9 (PMC2831852; doi:10.1186/1476-072X-9-9)

APPENDICES

Wang, et al.: National Variation in United States Sepsis Mortality: a Descriptive Study

Appendices 1A-1D – Age-stratified maps

Appendices 2A-2B – Sex-stratified maps

Appendices 3A-3G – Infection group-stratified maps

APPENDIX 1A

Age-stratified regional variation in sepsis-attributed mortality, United States, 1999-2005. Excludes Alaska and Hawaii. Bold borders indicate states where reliable mortality rates could not be calculated.

Ages 15-24:

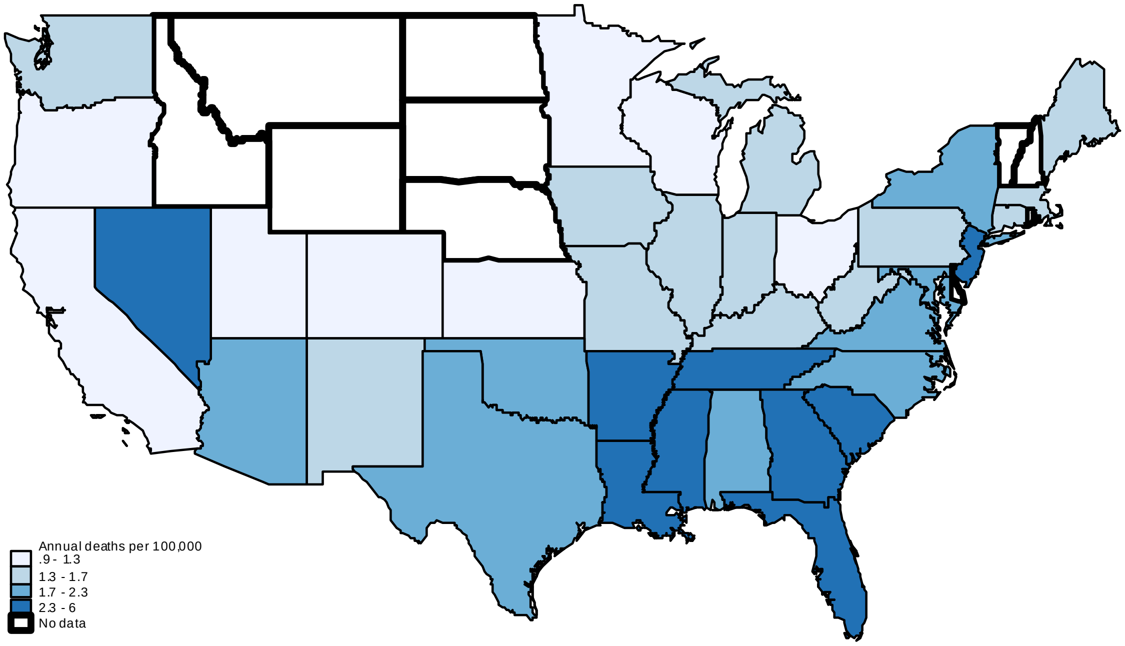

APPENDIX 1B

Age-stratified regional variation in sepsis-attributed mortality, United States, 1999-2005. Excludes Alaska and Hawaii.

Ages 25-44:

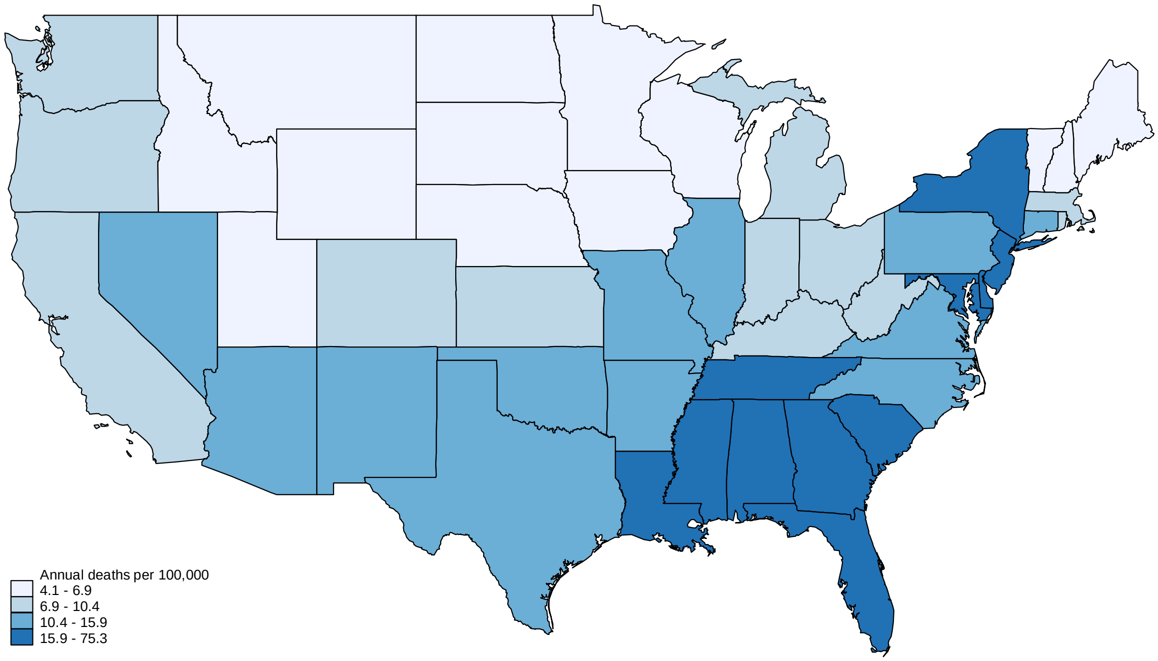

APPENDIX 1C

Age-stratified regional variation in sepsis-attributed mortality, United States, 1999-2005. Excludes Alaska and Hawaii.

Ages 45- 64

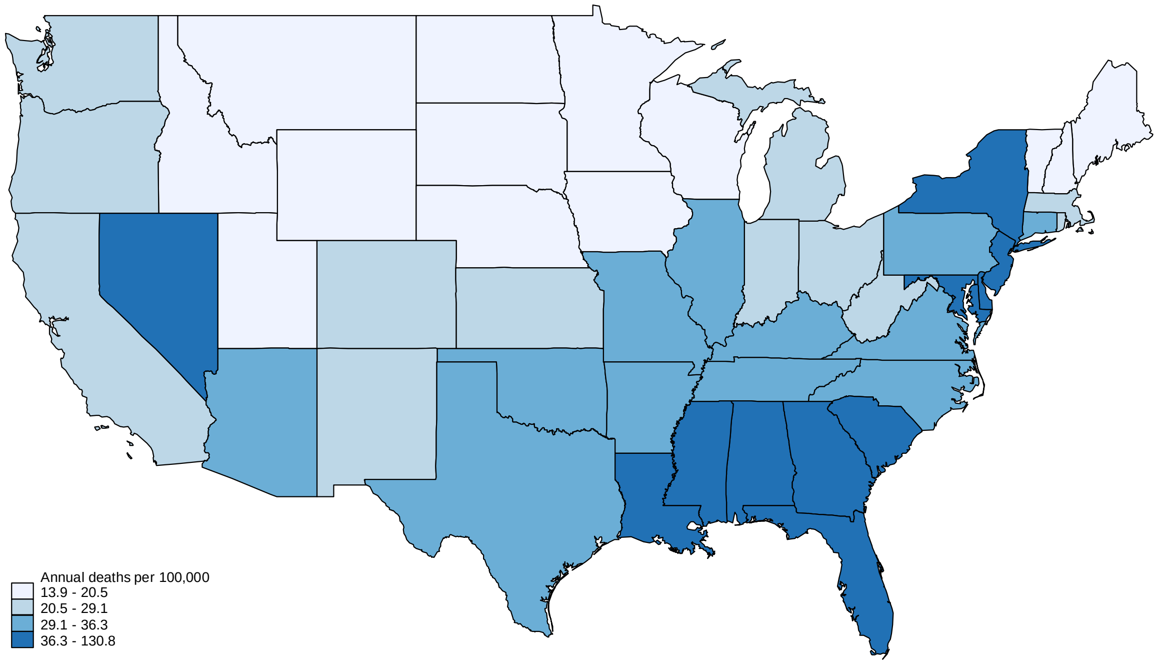

APPENDIX 1D

Age-stratified regional variation in sepsis-attributed mortality, United States, 1999-2005. Excludes Alaska and Hawaii.

Age >65:

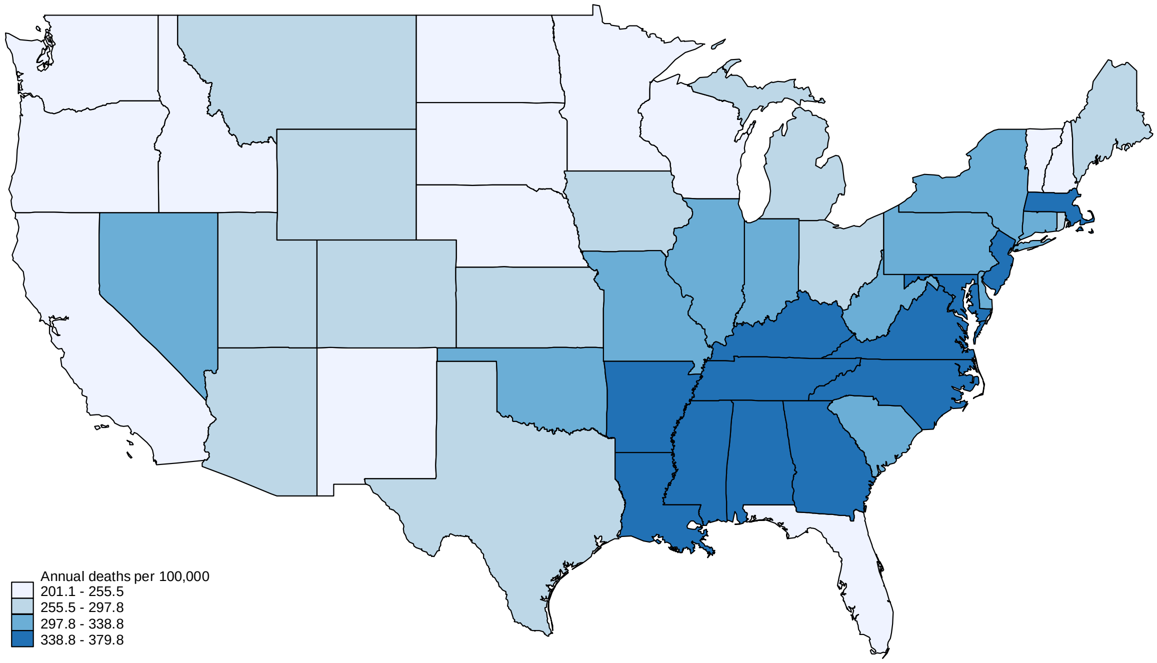

APPENDIX 2A

Regional variation in sepsis-attributed mortality, United States, 1999-2005 – males only. Excludes Alaska and Hawaii.

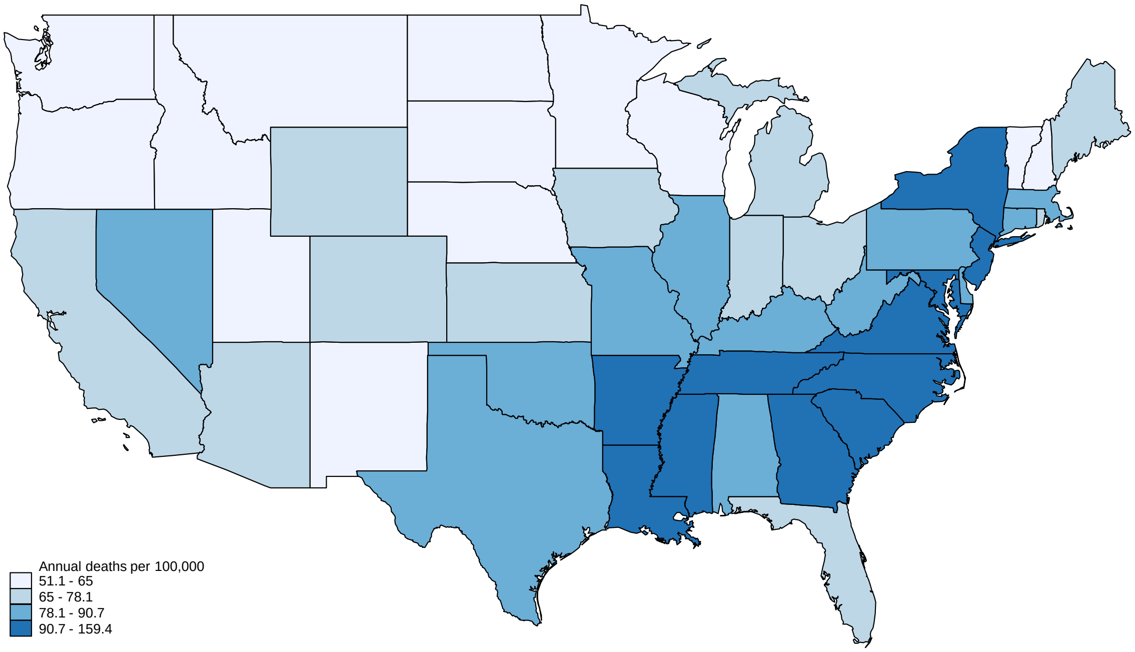

APPENDIX 2B

Regional variation in sepsis-attributed mortality, United States, 1999-2005 – females only. Excludes Alaska and Hawaii.

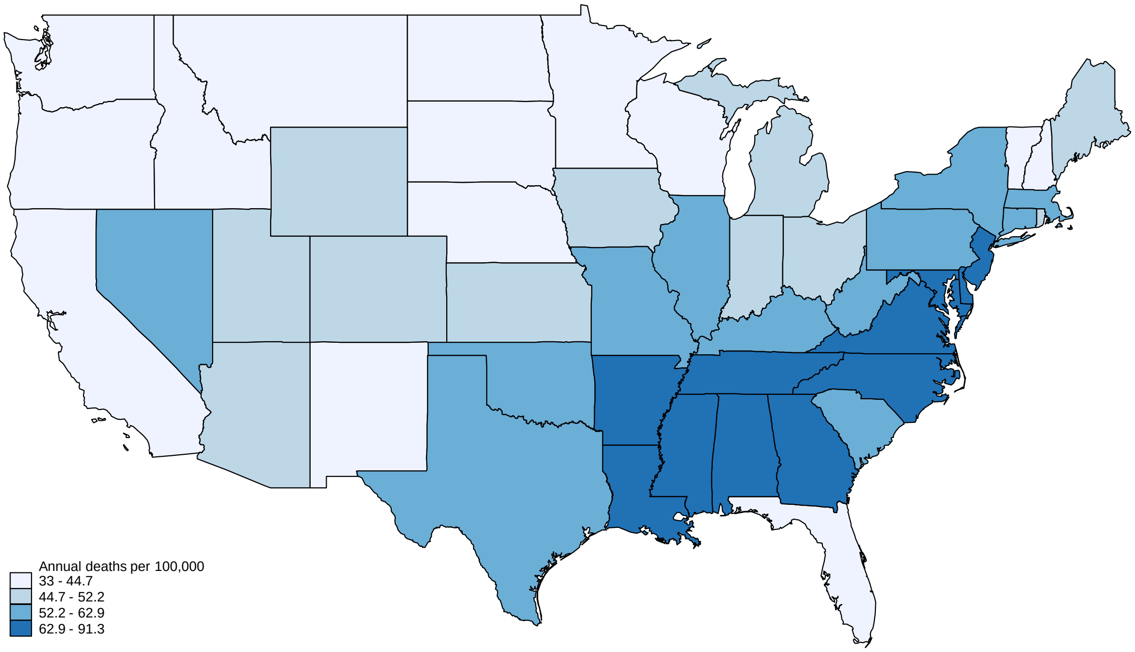

APPENDIX 3A

Regional variation in sepsis mortality – respiratory infections only, United States, 1999-2005. Excludes Alaska and Hawaii.

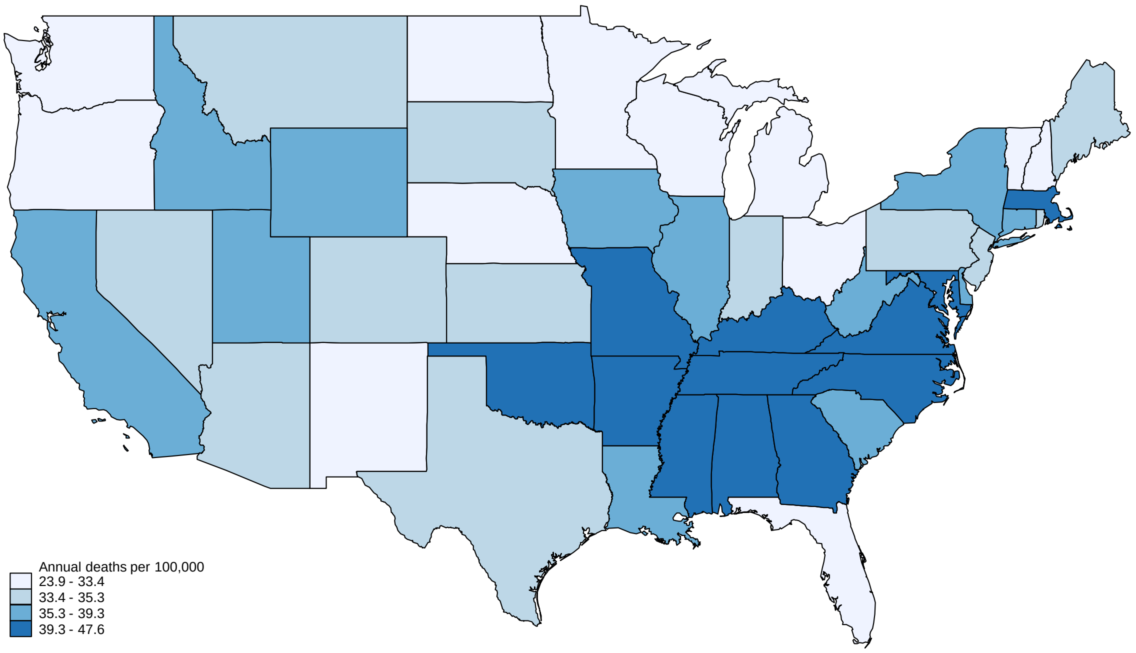

APPENDIX 3B

Regional variation in sepsis mortality – septicemia only, United States, 1999-2005. Excludes Alaska and Hawaii.

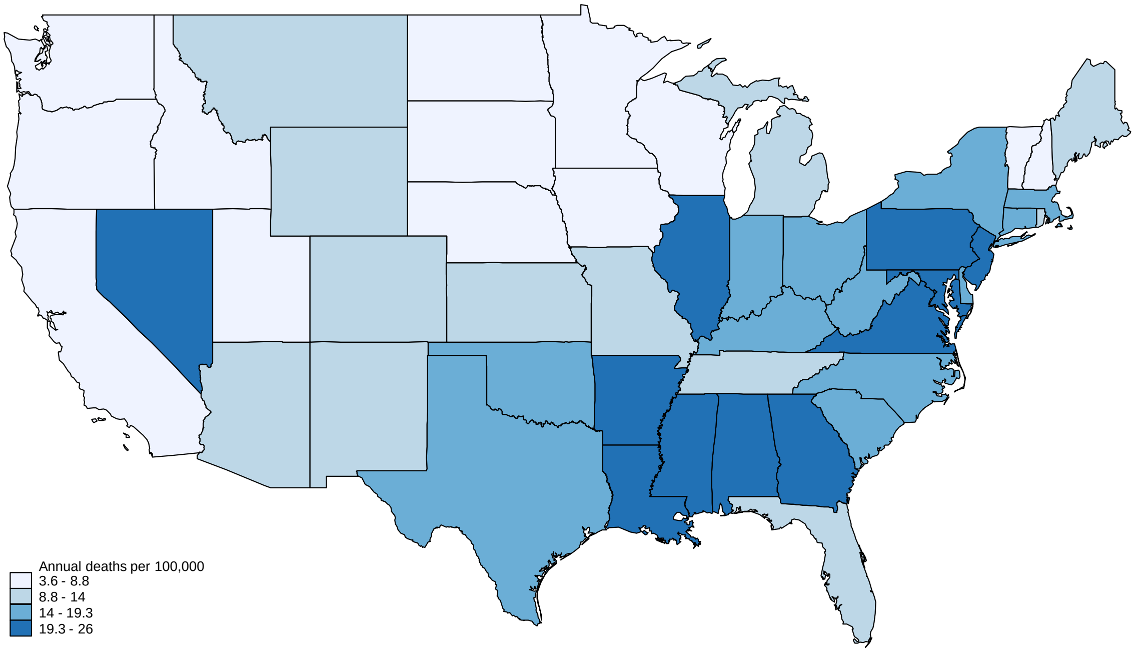

APPENDIX 3C

Regional variation in sepsis mortality – abdominal/gastrointestinal infections only, United States, 1999-2005. Excludes Alaska and Hawaii.

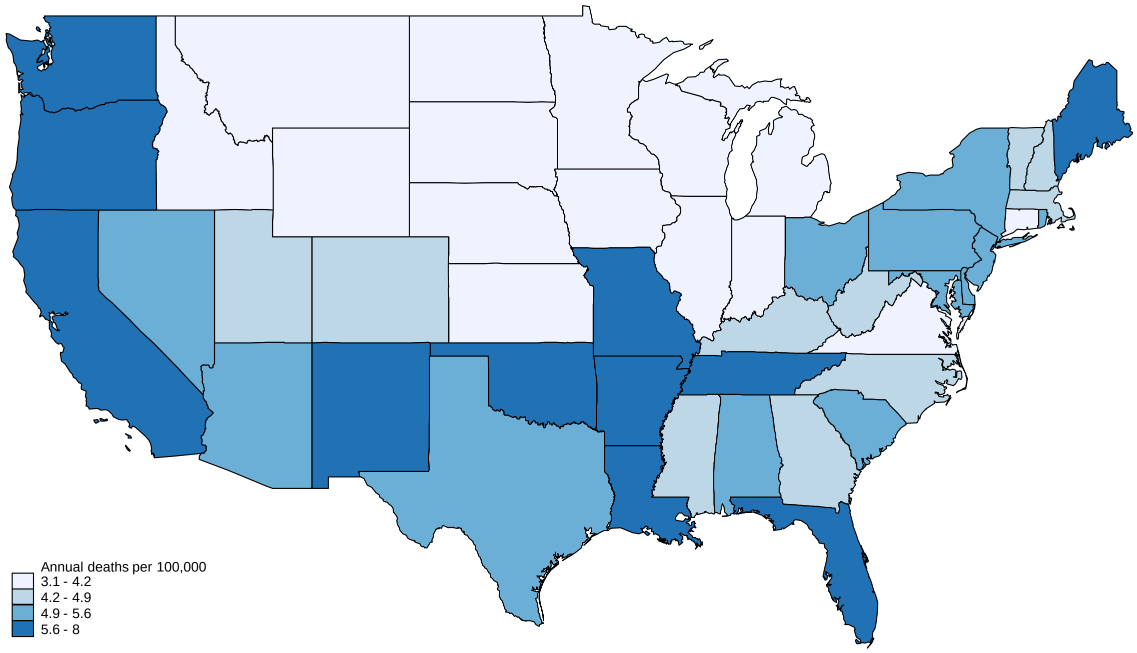

APPENDIX 3D

Regional variation in sepsis mortality – kidney/genitourinary infections only, United States, 1999-2005.

Excludes Alaska and Hawaii.

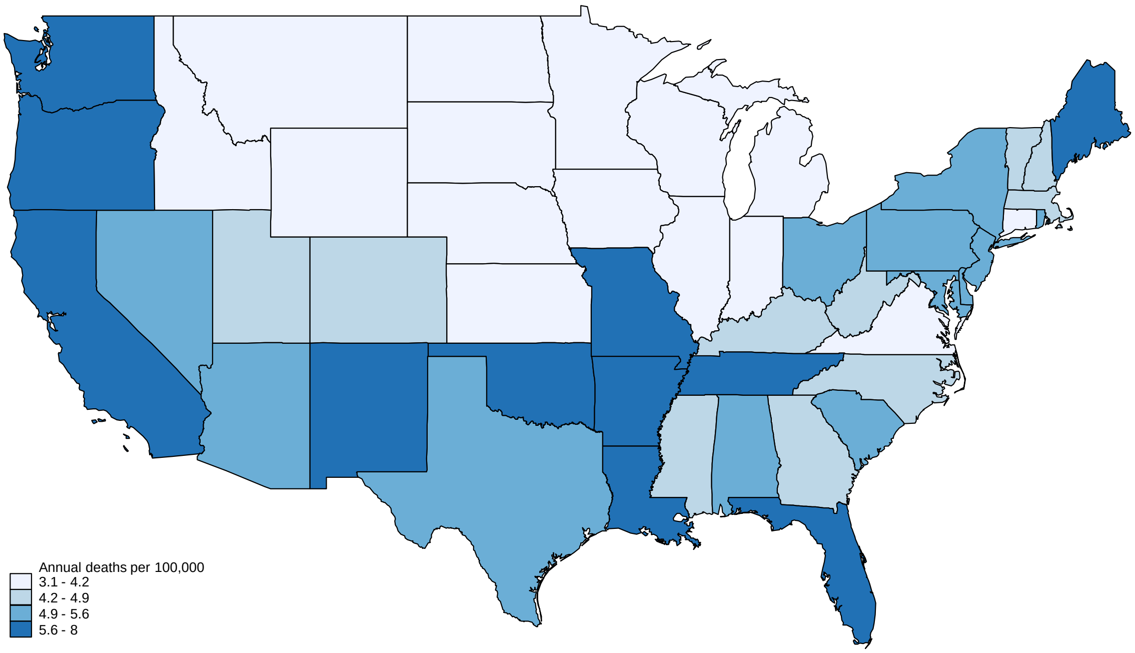

APPENDIX 3E

Regional variation in sepsis mortality – cardiac infections only, United States, 1999-2005. Excludes Alaska and Hawaii.

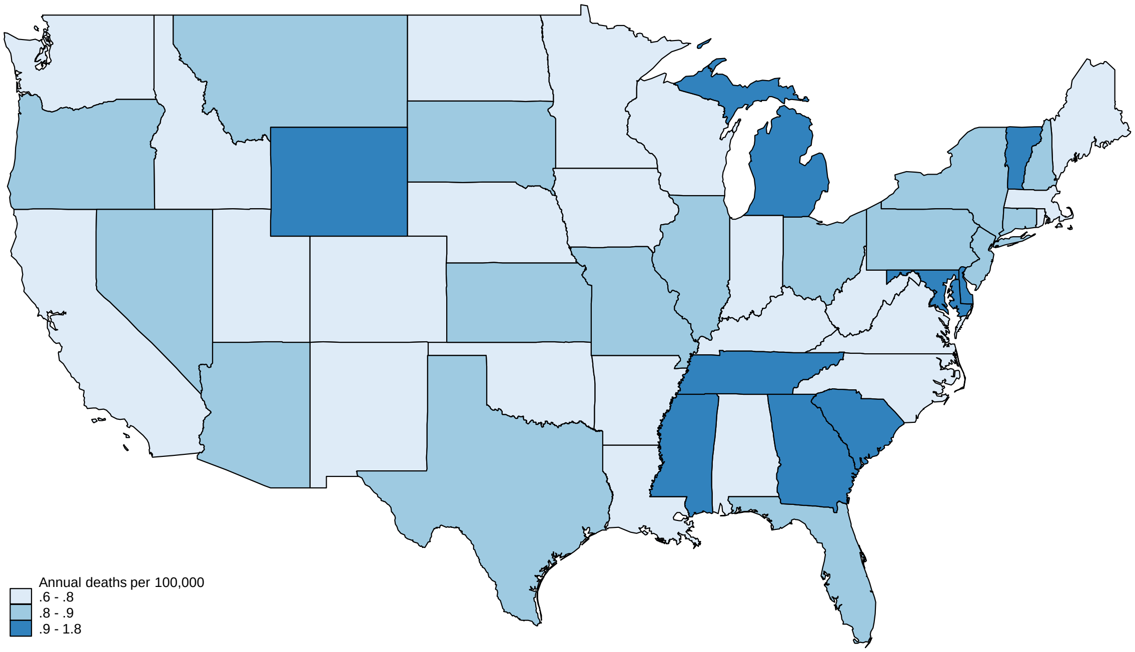

APPENDIX 3F

Regional variation in sepsis mortality – neurologic infections only, United States, 1999-2005. Excludes Alaska and Hawaii.

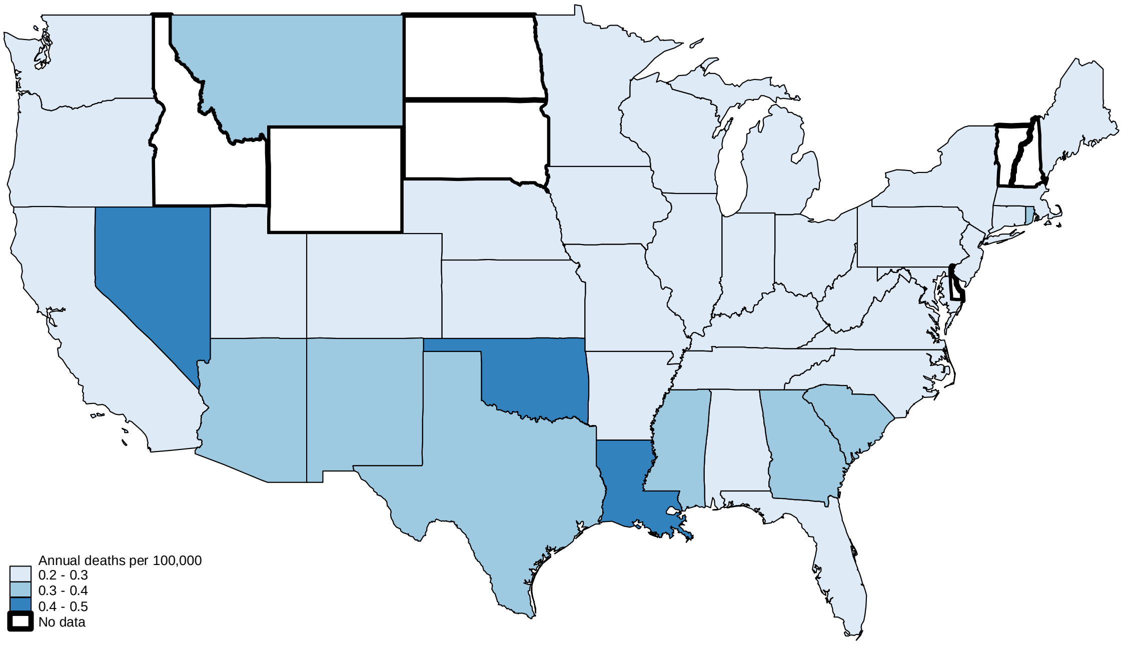

APPENDIX 3G

Regional variation in sepsis mortality – “other” infections only, United States, 1999-2005. Excludes Alaska and Hawaii.

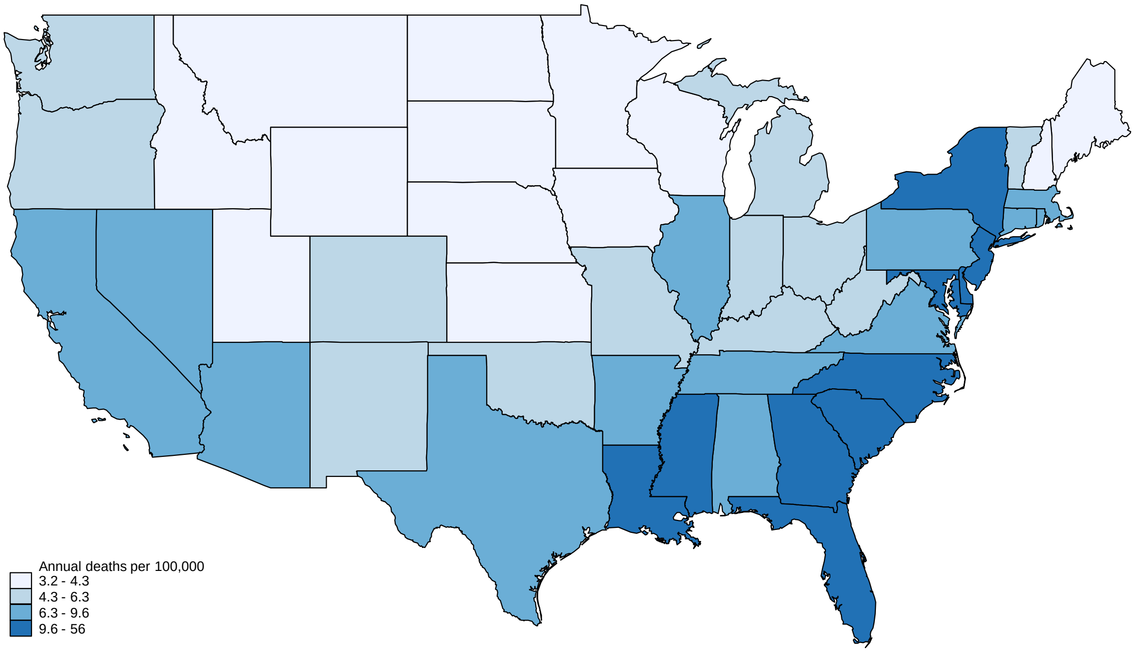

Supplement: Additional file 1 — APPENDICES, Wang, et al.: National Variation in United States Sepsis Mortality: a Descriptive Study. Appendices 1A-1D - Age-stratified maps; Appendices 2A-2B - Sex-stratified maps; Appendices 3A-3G - Infection group-stratified maps. [file 1476-072X-9-9-S1.PDF]
